# Identifying profile-specific candidate targets for miner safety: a latent class and network analysis of psychological resources

**DOI:** 10.3389/fpsyg.2026.1877732

**Published:** 2026-07-09

**Authors:** Xiaohua Pang, Jizu Li, Xuehua Xu

**Affiliations:** 1 School of Economics and Management, Taiyuan University of Technology, Taiyuan, China; 2 Department of Mental Health, Changzhi Medical College, Changzhi, China

**Keywords:** miner safety, psychological resources, latent class analysis, psychological network analysis, profile-specific intervention targets, person-job fit, knowledge sharing

## Abstract

**Introduction:**

Understanding how psychological resources configure to sustain miner safety is essential for designing targeted interventions, yet remains limited.

**Methods:**

Data were collected from 1,247 Chinese frontline miners who completed measures of transformational leadership, job crafting, work engagement, person-job fit, organizational commitment, knowledge sharing, and safety performance. Latent class analysis (LPA) was applied to identify psychological profiles, and Gaussian graphical models with EBICglasso regularization were estimated for each profile. Network centrality, bridge centrality, and a network-based candidate target index were computed to prioritize profile-specific intervention targets. Network Comparison Tests (NCT) were used to formally compare network structures across profiles.

**Results:**

Four profiles emerged, namely Job-Crafting-Driven (42.8%), Leadership-Dependent (11.4%), Low-Resource Vulnerable (18.8%), and Optimal (27.0%). Network density was highest in the Job-Crafting-Driven class (0.560) and lowest in the Optimal class (0.320). Person-job fit and knowledge sharing consistently ranked among the top three bridge nodes across all four profiles. The candidate target index revealed profile-specific priorities, such as person-job fit and knowledge sharing for the Job-Crafting-Driven group, and organizational commitment for the Low-Resource Vulnerable group. NCT indicated significant global strength differences between Class 1 and all other classes, and significant network structure differences between Classes 1 and 4, and between Classes 2 and 3.

**Discussion:**

These findings provide a foundation for generating testable hypotheses about profile-specific safety interventions, pending longitudinal validation. Practically, safety interventions should be tailored to distinct worker profiles, with person-job fit and knowledge sharing as priority targets for most profiles. Optimal profile centrality estimates were statistically unreliable and excluded from interpretation.

## Introduction

As a fundamental industry, the mining sector provides essential energy and raw materials yet remains universally recognized as high-risk. Miners work deep underground for extended periods, facing immediate hazards such as gas explosions, roof collapses, and coal dust exposure (Tian et al., 2024). Confined, poorly lit, noisy, and humid conditions, combined with irregular shift patterns, challenge miners’ physical health and psychological wellbeing, contributing to elevated stress, anxiety, and burnout (Li et al., 2024). Despite technological and institutional improvements, achieving “zero harm” continues to be a formidable challenge.

This reality has prompted a paradigm shift in safety science. Historically, safety management emphasized technocentric approaches, focusing on machinery and processes (Zohar, 2010). However, more than 80% of safety incidents are rooted in human factors (Heinrich et al., 1980). Researchers increasingly recognize that miners’ psychological states and organizational behaviors are deeper determinants of safety performance. Constructs such as work engagement, organizational commitment, and the quality of leader-subordinate interactions have emerged as pivotal predictors of safety compliance and proactive participation (Neal and Griffin, 2006). Consequently, safety is now understood as a complex socio-psychological system rather than a purely technical issue. Despite these conceptual advances, safety interventions in mining are typically designed and deployed as one-size-fits-all programs, with little consideration of individual differences in psychological resource profiles. This uniform approach may partially explain why many well-intentioned interventions yield modest or inconsistent results. Such interventions are not targeted at the specific nodes that drive the safety-related psychological system of a given subgroup of workers. Identifying which psychological resources are structurally central for different types of miners is therefore a prerequisite for designing more precise, resource-efficient interventions.

Against this backdrop, the present study examines the psychological and behavioral mechanisms underlying miner safety through an integrated multi-theoretical lens. Drawing upon the Job Demands-Resources (JD-R) model (Tummers and Bakker, 2021), social exchange theory (Birtch et al., 2016), and person-environment fit theory (Kristof-Brown et al., 2005), the study conceptualizes a sequential pathway from contextual inputs to individual regulation, internal psychological states, organizational behaviors, and safety outcomes. Seven constructs, transformational leadership, job crafting, work engagement, knowledge sharing, person-job fit, organizational commitment, and safety performance, are identified as core elements of this system.

### Transformational leadership and job crafting as contextual and proactive inputs

Transformational leadership constitutes a pivotal contextual input in safety-critical industries. Grounded in social exchange theory (Blau, 1964), leaders who demonstrate moral modeling, inspirational motivation, individualized consideration, and charisma foster climates of trust and psychological safety (Inceoglu et al., 2018; Li and Shi, 2005). Recent evidence confirms that safety-specific transformational leadership enhances compliance and proactive safety behaviors (Ghasemi et al., 2025; Zhang et al., 2022). While leadership sets the context, miners actively regulate their work through job crafting. Rooted in the JD-R model (Bakker and Demerouti, 2007) and proactive job design theory (Parker and Collins, 2010), job crafting reflects employees’ bottom-up efforts to align tasks with personal resources. In mining, job crafting manifests in increasing resources, increasing challenges, and reducing hindrances (Tims et al., 2012; Tims and Bakker, 2010). Recent studies highlight its role as a resilience-building mechanism (Munnisunker and Dhanpat, 2025).

### Internal psychological states

The effectiveness of leadership and job crafting is mediated through miners’ internal psychological states. Work engagement, characterized by vigor, dedication, and absorption (Schaufeli et al., 2002), represents the energetic dimension. Person-job fit reflects cognitive alignment between individual capabilities and job demands, serving as a stabilizing foundation (Kristof-Brown et al., 2005). Organizational commitment, particularly its affective dimension, arises from perceptions of organizational investment and fosters loyalty and collective responsibility (Kim et al., 2016; Curcuruto and Griffin, 2018).

### Knowledge sharing and safety performance

Safety in mining also depends on collective intelligence. Knowledge sharing, anchored in knowledge management theory (Nonaka and Takeuchi, 1995) and social capital theory (Nahapiet and Ghoshal, 1998), represents the behavioral bridge connecting individual expertise with organizational learning. Tacit knowledge, such as recognizing early signs of equipment failure, is particularly critical (Collins and Smith, 2006). Employees embedded in climates of trust and engagement are more willing to share knowledge (Wang and Noe, 2010). Safety performance, the ultimate criterion, is defined through safety compliance and safety participation (Neal and Griffin, 2004; Christian et al., 2009). Recent studies link it to leadership, job crafting, engagement, commitment, and knowledge sharing (Lei et al., 2024; Zhu et al., 2022).

### Theoretical foundations of psychological network analysis

Network theory posits that systems consist of interdependent elements, and that the essential properties of a system derive from the patterns of interaction among them (Borsboom, 2017). This perspective shifts the research focus from variables themselves to the structure of relationships between variables, transcending traditional variable-centered approaches. Conceptualizing the seven constructs in this study as a causal system is theoretically justified, given the bidirectional and mutually reinforcing relationships among them (van der Maas et al., 2006).

Psychological network analysis offers several methodological advantages for the present investigation. Centrality analysis can identify precise intervention targets (Robinaugh et al., 2016), bridge centrality can reveal critical influence pathways connecting subsystems (Jones et al., 2021), and simulation-based intervention algorithms can model the hypothesized propagation effects of targeting specific nodes on the entire network (Zhang et al., 2025). Furthermore, LCA combined with network comparison tests (NCT) enables the identification of distinct psychological profiles and their corresponding network architectures, moving beyond the assumption of population homogeneity (van Borkulo et al., 2023).

Although the JD-R model, social exchange theory, and person-environment fit theory originated independently, they converge on a common premise: psychological resources and social exchanges do not operate in isolation but form interconnected systems. The JD-R model provides the foundational distinction between contextual inputs (leadership, job crafting) and psychological states (engagement, fit). Social exchange theory adds the mechanism of reciprocity, through which supportive leadership and organizational commitment become mutually reinforcing. Person-environment fit theory highlights the stabilizing role of perceived congruence, which serves as a meta-resource facilitating the mobilization of other resources. Integrating these three frameworks within a network model allows us to test how these theoretically distinct constructs interrelate as a system, rather than treating them as isolated predictors in separate regression equations. The network approach, with its capacity to identify central hubs, bridge nodes, and profile-specific architectures, is uniquely suited to capture this systemic organization.

### The present study

Recent work has begun to integrate person-centered profiling with network estimation in occupational health contexts. Portoghese et al. (2025) applied latent profile analysis followed by profile-specific network analysis to occupational health variables among Italian hospital workers, identifying density gradients across profiles and demonstrating that network structures differed systematically across latent profiles. Similarly, Zhao et al. (2026) recently employed EBICglasso network estimation with bridge centrality analysis among Chinese firefighters to identify cross-system bridge variables linking psychological distress and resilience. The present study extends this emerging methodological approach in two respects. First, by applying LCA-based network analysis to the high-risk mining context, it tests the generalizability of profile-specific network heterogeneity beyond healthcare and emergency service populations. Second, and more substantively, it explicitly computes a network-based candidate target index that integrates centrality and bridge strength to prioritize profile-specific candidate intervention targets, an emphasis absent from prior LPA–network integration studies, which have focused primarily on describing network differences rather than translating them into intervention priorities. Despite the proliferation of research on individual predictors of safety behavior, three critical gaps remain. First, few studies have examined how the core psychological and behavioral constructs of miner safety operate as an interconnected system rather than as isolated variables. Second, it is unknown whether distinct psychological profiles exist among miners that are characterized by qualitatively different network structures and, hence, require different intervention strategies. Third, no study to date has employed simulation-based network analysis to identify profile-specific candidate intervention-relevant nodes for miner safety.

To address these gaps, the present study pursued three objectives. First, we used LCA to identify distinct psychological profiles among 1,247 Chinese frontline miners based on 15 dimensions spanning transformational leadership, job crafting, work engagement, person-job fit, organizational commitment, knowledge sharing, and safety performance. LCA was selected over latent profile analysis because the 15 dimensions were discretized into three categories (low, medium, high) to facilitate interpretation and to reduce the influence of distributional assumptions; LCA was selected over cluster analysis because it provides model-based fit statistics (e.g., BIC, entropy) for determining the optimal number of profiles. Second, we estimated a Gaussian graphical model within each latent class and computed centrality, bridge centrality, and an integrated network-based candidate target index to identify profile-specific candidate intervention priorities. Third, we employed network comparison tests to formally compare the network architectures across the identified profiles.

We addressed the following research questions:

RQ1: What distinct psychological profiles can be identified among frontline miners based on the seven core constructs and their constituent dimensions?

RQ2: Which nodes serve as central hubs, bridge connectors, and high-priority intervention targets within each profile-specific network?

RQ3: How do network structures and global connectivity differ across the identified profiles?

## Methods

### Participants

This study was conducted in June 2025 at three state-owned intelligent coal mines in Shanxi Province, China. Participants were frontline miners who provided informed consent. Exclusion criteria included non-frontline roles or unwillingness to participate. A total of 1,500 questionnaires were distributed across the three mines. After removing responses that were incomplete (missing >20% of items), completed in less than 3 min, or showed patterned answering (e.g., same response for >90% of items), 1,247 valid responses were retained, yielding an effective response rate of 83.1%. Missing values for retained cases (<5% per item) were imputed using median substitution. Participants ranged in age from 19 to 60 years, with 281 (22.5%) younger than 35, 602 (48.3%) between 35 and 45, and 364 (29.2%) older than 45.

### Research instruments

Mature and widely adopted scales were used to measure the seven core constructs and their constituent dimensions. All items were rated on a five-point Likert scale (1 = strongly disagree to 5 = strongly agree), with the exception of organizational commitment, which employed a six-point scale. The sources, dimensions, sample items, and reliability coefficients are summarized in Table 1. Cronbach’s *α* values, calculated from the present sample, ranged from 0.920 to 0.977.

**Table 1:** Summary of measurement scales and their reliabilities.

Variable	Scale and source	Dimension	Sample item	No. of items	Cronbach’s *α*
Transformational leadership	Transformational Leadership Questionnaire (TLQ) (Li and Shi, 2005)	Moral modeling	Is incorruptible and selfless.	8	0.960
		Inspirational motivation	Points out goals and direction for employees.	6	0.968
		Leadership charisma	Has strong professional competence.	6	0.973
		Individualized consideration	Helps employees with life and family difficulties.	6	0.973
Job crafting	Job Crafting Scale (Tims et al., 2012)	Increasing structural job resources	I try to develop my capabilities.	5	0.968
		Increasing social job resources	I ask my supervisor to coach me.	5	0.946
		Increasing challenging job demands	When an interesting project comes along, I offer myself proactively.	5	0.954
		Decreasing hindering job demands	I make sure that my work is mentally less intense.	6	0.955
Work engagement	Utrecht Work Engagement Scale (UWES) (Schaufeli et al., 2006)	Vigor	At my work, I feel bursting with energy.	3	0.959
		Dedication	I am enthusiastic about my job.	3	0.972
		Absorption	I am immersed in my work.	3	0.968
Organizational commitment	Organizational Commitment Scale [Mowday et al., 1979; Adapted by Ling et al. (2000)]	Affective commitment (unidimensional in this study)	1. I am proud to tell others that I am part of this company. 6. I feel a strong sense of belonging to this company.	8	0.951
Person-Job Fit	Person-Job Fit Scale [Adapted from Singh and Greenhaus (2004)]	Global fit	I feel a strong match between myself and my current job.	4	0.960
Knowledge sharing	Knowledge Sharing Scale (Collins and Smith, 2006; Tian, 2015)	Knowledge sharing willingness	I am willing to share knowledge with my colleagues.	3	0.958
		Knowledge sharing behavior	We can skillfully solve work problems by exchanging and combining knowledge.	4	0.966
Safety performance	Safety performance scale [Adapted from Neal and Griffin (2006)]	Safety compliance	I use all necessary safety equipment.	3	0.920
		Safety participation	I proactively participate in safety training.	3	0.850

### Statistical analysis

All analyses were conducted in R (Version 4.5.1; R Core Team, 2021). Key packages included poLCA (v1.4.1) for latent class analysis, bootnet (v1.5.5) and qgraph (v1.9.5) for network estimation and visualization, networktools (v1.5.1) for bridge centrality, and NetworkComparisonTest (v2.2.1) for network comparisons.

#### Data preprocessing

**Figure 1:**
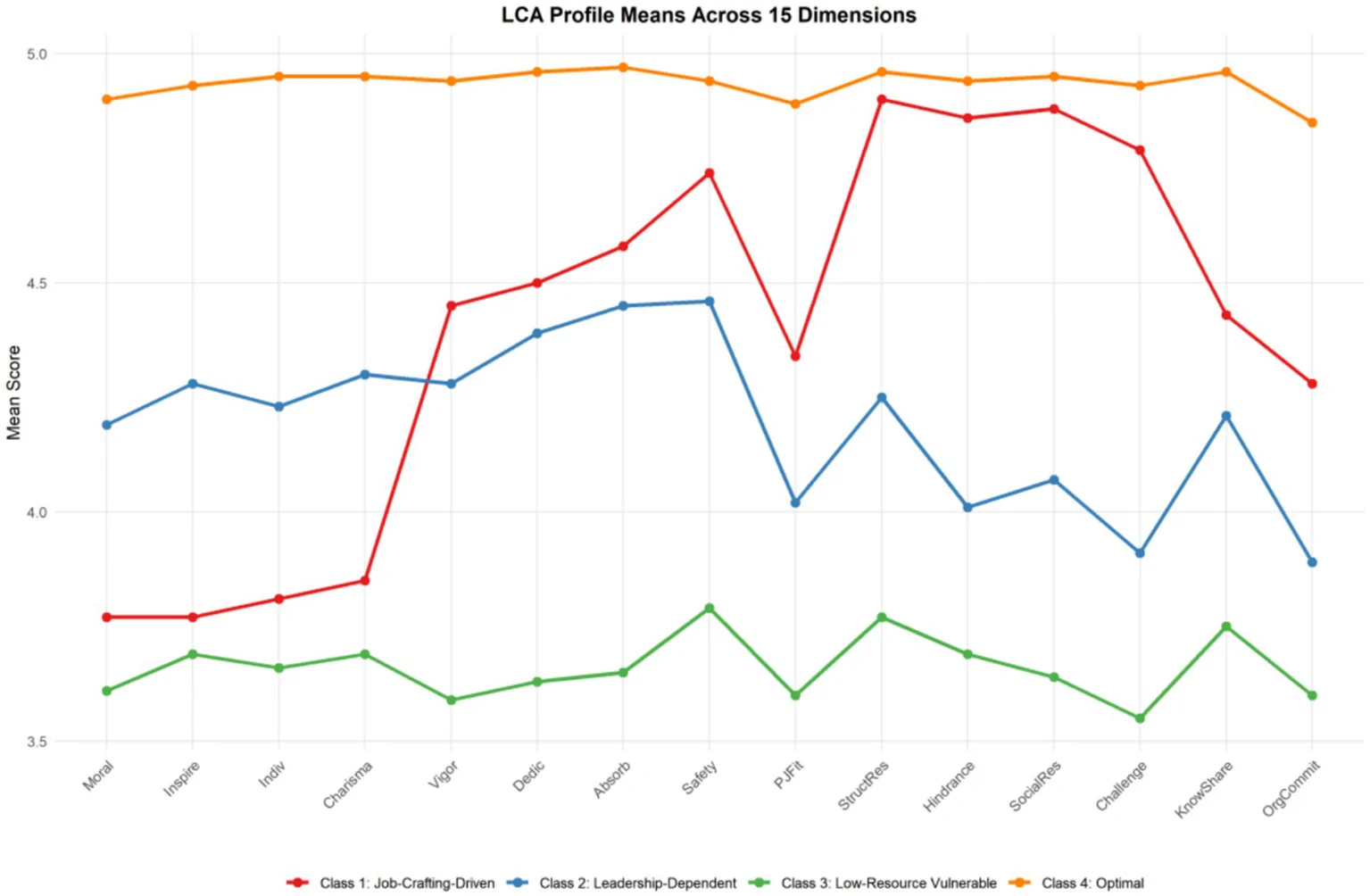
LCA profile means across 15 dimensions.

The 15 continuous dimension scores were discretized into three categories (low, medium, high) using the 33rd and 67th percentiles as cut-points for each dimension. Discretization was adopted for two reasons. First, it facilitates substantive interpretation of the latent classes by mapping each dimension onto an intuitive low-medium-high framework that is readily communicable to practitioners. Second, it reduces the influence of distributional assumptions inherent in latent profile analysis, which assumes continuous indicators follow a multivariate normal distribution. Because organizational commitment was measured on a six-point Likert scale while all other constructs used a five-point scale, organizational commitment scores were rescaled to a five-point metric using the formula OC₅pt = 1 + (OC₆pt − 1) × 4/5, which preserves the original scale endpoints (1–5). LCA was then performed on these discretized categorical indicators. Figure 1 presents the original continuous means for each latent class to facilitate substantive interpretation of the profiles. It should be noted that discretization may result in some information loss; however, this trade-off was considered acceptable given the enhanced interpretability and reduced distributional assumptions.

#### Latent class analysis

To identify distinct profiles of miners’ psychological and behavioral characteristics, we performed LCA on the 15 dimension scores. Models with two through six classes were estimated. The optimal number of classes was determined by the Bayesian Information Criterion (BIC) and the Elbow rule, balancing statistical parsimony with the interpretability and sample sizes of the resulting profiles.

#### Network estimation

A Gaussian graphical model (GGM) was estimated using the graphical least absolute shrinkage and selection operator (GLASSO) implemented in the mgm package (Haslbeck and Waldorp, 2020), combined with the Extended Bayesian Information Criterion (EBIC; tuning parameter *γ* = 0.5). The tuning parameter γ was set to 0.5, which is the default value recommended for psychological network analysis and balances sensitivity and specificity in edge detection (Epskamp and Fried, 2018). This value is widely adopted in prior psychological network research when the goal is to identify a parsimonious yet informative network structure. To evaluate the robustness of the network structures to this choice, we re-estimated all four class-specific networks using *γ* = 0.25 (more edges) and *γ* = 0.75 (fewer edges). The pattern of bridge centrality rankings was largely unchanged across *γ* values, with person-job fit and knowledge sharing consistently ranking among the top three bridge nodes. Detailed results are available in Supplementary Table S1. The resulting networks were visualized using the qgraph package (Epskamp et al., 2012). With 15 nodes and 105 possible edges, the ratio of observations to parameters is relatively low for the smaller classes, particularly Class 2 (*n* = 142). However, the use of regularization via GLASSO mitigates this concern to some extent by shrinking spurious edges to zero. Furthermore, the CS-coefficients for expected influence in Class 2 met acceptable thresholds (CS-EI = 0.514 > 0.50), supporting the interpretability of the centrality estimates. Within each latent class, GGMs were estimated using the original continuous dimension scores (i.e., prior to discretization), as GGM assumes continuous variables. All variables were standardized within each class prior to network estimation. Edges represent regularized partial correlations estimated via the graphical LASSO with EBIC model selection (*γ* = 0.5).

#### Role of safety performance

We acknowledge that safety performance is conceptualized as the outcome variable in the theoretical framework yet is also included as a node in the LCA and network estimation. This decision was made because the network perspective treats all psychological and behavioral variables as mutually interacting nodes within a system, rather than assigning them *a priori* directional roles. Including safety performance in the profile construction and network estimation is therefore consistent with the systemic perspective adopted in this study. To assess the robustness of our findings to this modeling choice, we conducted sensitivity analyses excluding safety performance from the LCA and network estimation. The resulting four profiles closely mirrored those from the original analysis. After accounting for the reordering of class labels across analyses, classification consistency reached 95.0%, indicating that the inclusion of safety performance did not substantively alter profile membership. Moreover, the bridge centrality patterns were replicated in the sensitivity analysis, with person–job fit and knowledge sharing remaining the top bridge nodes in the corresponding profiles.

#### Centrality and bridge centrality

Strength, closeness, betweenness, and expected influence (EI) were computed for each class-specific network using qgraph and bootnet. EI, which accounts for the sign of edge weights, was used as the primary centrality metric because all edges were positive. Bridge strength was calculated using networktools to identify nodes that connect predefined communities: transformational leadership (4 nodes), work engagement (3 nodes), safety performance (1 node), person-job fit (1 node), job crafting (4 nodes), knowledge sharing (1 node), and organizational commitment (1 node).

#### Network-based candidate target index

To prioritize intervention targets within each profile, we computed a network-based candidate target index by averaging the standardized scores of EI and bridge strength for each node. This index was created for the present study. These two metrics were selected because they capture complementary aspects of a node’s structural position. EI reflects a node’s direct connections within its immediate neighborhood, whereas bridge strength reflects its role in connecting different communities. Equal weighting was adopted as a parsimonious default, consistent with the practice of averaging standardized indices in composite measures where no strong *a priori* rationale exists for differential weighting. Sensitivity analyses using alternative weightings yielded highly consistent rankings, supporting the robustness of the equal-weighting approach. Integrating both metrics yields a measure that identifies nodes with the greatest potential to be candidate targets for intervention research, as their structural position suggests hypothetical system-wide benefits.

#### Network accuracy and stability

Edge-weight accuracy was assessed using a nonparametric bootstrap with 5,000 samples. The stability of centrality indices was evaluated via case-dropping bootstrap, reporting correlation-stability coefficients (CS-coefficients); values above 0.50 indicate strong stability, while values above 0.25 are considered acceptable.

#### Network comparison across latent classes

To formally test whether network structures differed between the four identified profiles, we conducted pairwise Network Comparison Tests (NCT) with 500 permutations. The NCT evaluates differences in global strength and overall network structure.

#### Common method bias

Because all constructs were measured via single-wave self-report questionnaires, we assessed common method bias (CMB). Although Harman’s single-factor test is widely reported, it has been criticized for being overly sensitive and for conflating genuine construct covariance with method variance, particularly in multidimensional wellbeing measures where a dominant general factor is theoretically expected (Podsakoff et al., 2003; Fuller et al., 2016). We therefore relied on two more rigorous procedures. First, confirmatory factor analysis (CFA) comparing a single-factor model to a six-factor model (A six-factor model was used because the 15 dimensions were assigned to six broader factors, with safety performance excluded as the outcome variable) showed that the single-factor model fit the data poorly, *χ*^2^(135) = 8,104.52, CFI = 0.703, RMSEA = 0.218, whereas the six-factor model demonstrated good fit, *χ*^2^(120) = 710.67, CFI = 0.978, RMSEA = 0.063. The six-factor model significantly outperformed the single-factor model (ΔCFI = 0.275, exceeding the recommended 0.01 threshold; Cheung and Rensvold, 2002). Second, we compared standardized factor loadings between the six-factor model and a model including an orthogonal latent method factor. The average change in loadings was 0.023 (SD = 0.018), with a maximum change of 0.041. No indicator’s loading changed by more than 0.05, indicating that the method factor did not substantially alter the item-construct relationships. These results collectively indicate that common method bias is unlikely to threaten the validity of the findings.

## Results

### Descriptive statistics

The final sample comprised 1,247 frontline miners (88.9% male), with 281 (22.5%) younger than 35 years, 602 (48.3%) between 35 and 45 years, and 364 (29.2%) older than 45 years. Descriptive statistics for the full sample are reported in Table 2. However, given that the latent class is the primary unit of analysis, readers are directed to Figure 1 for the profile-level means, which are more directly informative for the substantive interpretation of the profiles. All scales demonstrated acceptable to excellent internal consistency (Cronbach’s *α* = 0.921–0.977; see Table 1 for full list).

**Table 2:** Means, standard deviations, and internal consistency of all dimensions (*N* = 1,247).

Dimension	*M*	SD	*α*
Moral modeling	4.09	0.81	0.960
Inspirational motivation	4.14	0.76	0.968
Individualized consideration	4.13	0.76	0.973
Leadership charisma	4.16	0.73	0.973
Vigor	4.18	0.82	0.959
Dedication	4.23	0.82	0.972
Absorption	4.27	0.79	0.968
Safety performance	4.33	0.73	0.921
Person-job fit	4.11	0.77	0.960
Structural resources	4.31	0.69	0.955
Reducing hindrances	4.22	0.72	0.946
Social resources	4.21	0.75	0.954
Challenge demands	4.13	0.80	0.955
Knowledge sharing	4.24	0.67	0.977
Organizational commitment	4.07	0.75	0.951

M, mean; SD, standard deviation; α, Cronbach’s alpha.

### Latent class analysis

LCA was performed on the 15 psychological dimensions. Models with two through six classes were estimated, and their fit indices are displayed in Table 3. Model selection was guided by multiple fit indices, including AIC, BIC, sample-size adjusted BIC (aBIC), entropy, and the Lo–Mendell–Rubin (LMR) likelihood ratio test. All models showed excellent class separation, with entropy values ranging from 0.939 to 0.971. The LMR test was significant at each step from two through six classes (all *p* < 0.001), indicating that each additional class significantly improved model fit. However, the four-class solution was retained for three reasons. First, the ΔBIC from four to five classes was relatively modest (283.80) compared to the preceding decrease from three to four classes (591.57), suggesting diminishing returns in model improvement; aBIC followed the same pattern. Second, the four-class model maintained high classification quality, with entropy = 0.958 and average posterior class probabilities of 0.957 (Class 1), 0.948 (Class 2), 0.988 (Class 3), and 0.993 (Class 4). Third, the four-class solution yielded substantively interpretable and clearly differentiated profiles, with each class exceeding 140 participants and meeting the minimum sample size recommendations for regularized network estimation. To evaluate the robustness of the findings to the class enumeration decision, the five-class solution was also examined. The five-class model yielded a smaller additional subgroup (*n* = 113) that was conceptually similar to one of the four main classes and did not alter the substantive interpretation of the profiles. The key bridge centrality patterns were replicated in the five-class solution.

**Table 3:** LCA model fit indices for two- to six-class solutions.

Classes	AIC	BIC	aBIC	ΔBIC	Entropy	LMR *p*
2	18,556.34	18,797.38	18,648.09	—	0.971	—
3	16,174.61	16,538.73	16,313.21	2,258.65	0.964	<0.001
4	15,459.95	15,947.16	15,645.39	591.57	0.958	<0.001
5	15,053.07	15,663.36	15,285.36	283.80	0.939	<0.001
6	14,806.49	15,539.86	15,085.63	123.50	0.944	<0.001

BIC, Bayesian Information Criterion; aBIC, sample-size adjusted BIC; ΔBIC, improvement in BIC from the k-class to the (k + 1)-class solution; LMR, Lo–Mendell–Rubin likelihood ratio test. The four-class solution (bolded) was selected based on the Elbow rule, aBIC, LMR, and profile interpretability. Entropy values range from 0 to 1, with values > 0.80 indicating good class separation.

Based on the Elbow rule and interpretability, a four-class model was retained (BIC = 15,947.2). Figure 1 displays the mean dimension scores for each class. Class 1 (Job-Crafting-Driven, *n* = 534, 42.8%) was distinguished by elevated job crafting scores, particularly structural resource seeking (*M* = 4.90), hindrance reduction (*M* = 4.86), and social resource seeking (*M* = 4.88). Class 2 (Leadership-Dependent, *n* = 142, 11.4%) showed the highest transformational leadership scores among non-optimal classes (e.g., inspirational motivation *M* = 4.28, leadership charisma *M* = 4.30). Class 3 (Low-Resource Vulnerable, *n* = 234, 18.8%) scored lowest across all dimensions (all Ms. < 4.0), though safety performance was relatively preserved (*M* = 3.79). Class 4 (Optimal, *n* = 337, 27.0%) showed near-ceiling functioning (Ms = 4.85–4.97).

### Network structures across LCA classes

**Figure 2:**
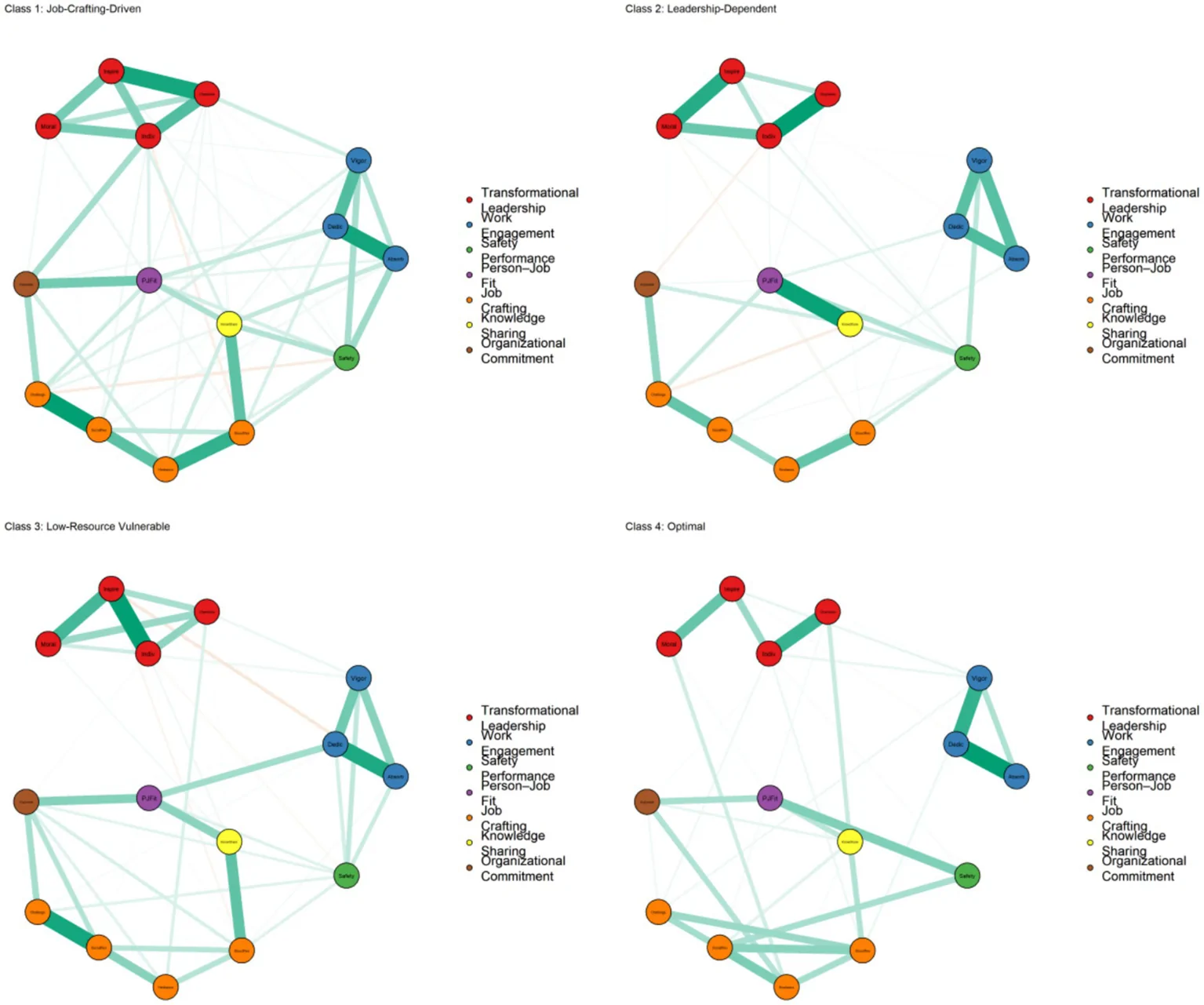
Estimated networks for the four LCA classes.

Separate GGMs were estimated within each class using EBICglasso (*γ* = 0.5). Figure 2 displays the four networks, and Table 4 summarizes their global properties. Network density decreased as resource levels increased, with values of 0.560 (Class 1), 0.364 (Class 2), 0.391 (Class 3), and 0.320 (Class 4). Non-zero edges followed the same pattern (63, 41, 44, 36, see Table 4).

**Table 4:** Network properties by LCA class.

Property	Class 1 (*n* = 534)	Class 2 (*n* = 142)	Class 3 (*n* = 234)	Class 4 (*n* = 337)
Density	0.560	0.364	0.391	0.320
Non-zero edges	63	41	44	36
Modularity (Q)	0.398	0.549	0.507	0.512

Modularity values from Louvain community detection. Higher Q indicates clearer community structure.

Regarding node centrality (Table 5), the most central nodes by Expected Influence varied. In Class 1, leadership charisma (EI = 1.372) and social resources (EI = 1.196) were most central; in Class 2, individualized consideration (EI = 1.753) and person-job fit (EI = 1.365); in Class 3, dedication (EI = 1.845) and social resources (EI = 1.640); in Class 4, social resources (EI = 1.635) and dedication (EI = 1.514).

**Table 5:** Top 5 nodes by expected influence in each LCA class.

Rank	Class 1	EI	Class 2	EI	Class 3	EI	Class 4	EI
1	Leadership charisma	1.372	Indiv. consideration	1.753	Dedication	1.845	Social resources	1.635
2	Social resources	1.196	Person-job fit	1.365	Social resources	1.640	Dedication	1.514
3	Reducing hindrances	0.880	Vigor	0.819	Inspirational motivation	1.186	Structural resources	1.085
4	Indiv. consideration	0.779	Inspirational motivation	0.414	Challenge demands	0.646	Reducing hindrances	1.025
5	Inspirational motivation	0.685	Moral modeling	0.407	Indiv. consideration	0.467	Vigor	0.568

### Bridge centrality

**Figure 3:**
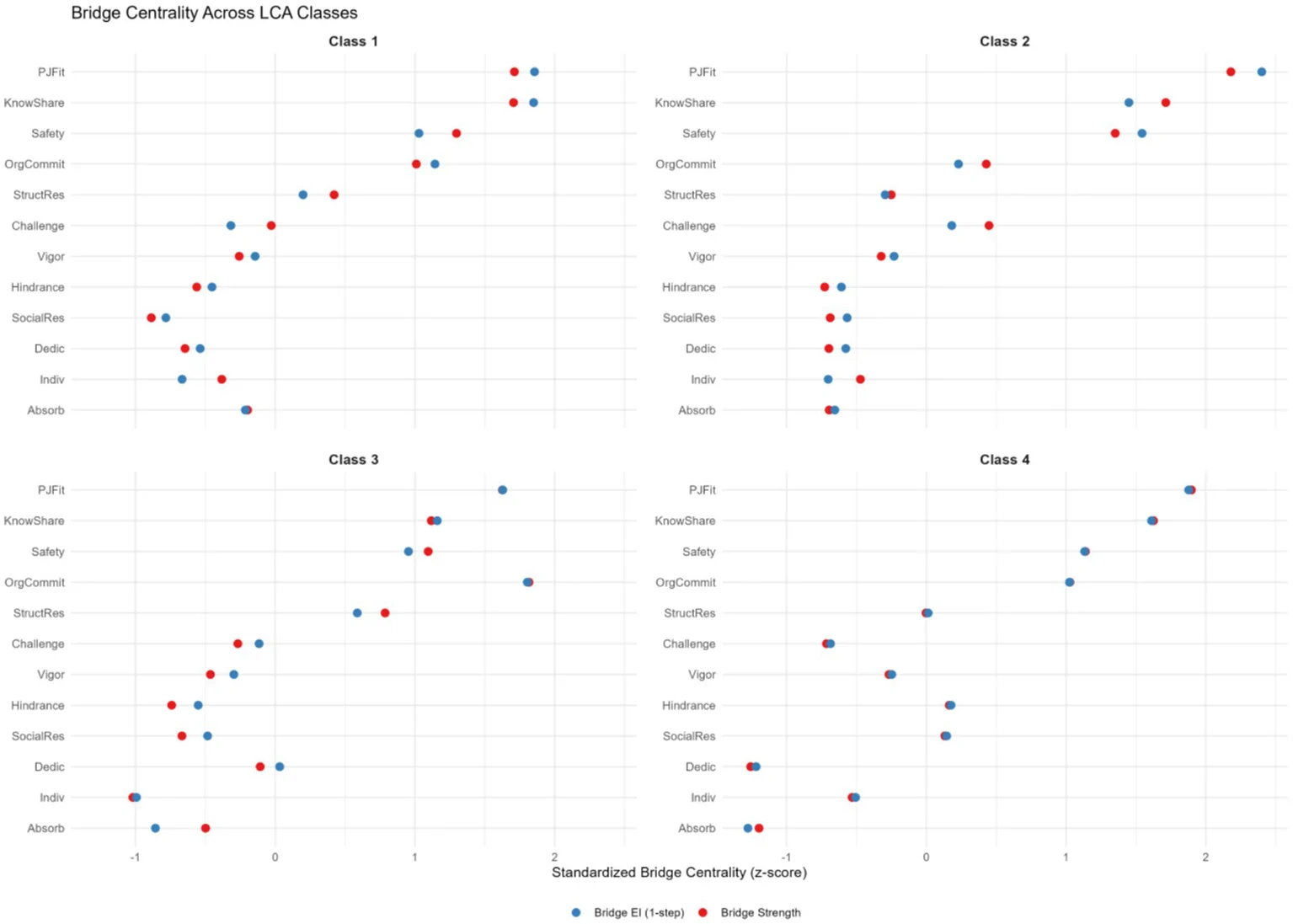
Bridge centrality comparison across LCA classes.

Bridge centrality identified nodes connecting different psychological communities (Jones, 2022) (Table 6). Person-job fit ranked as the strongest bridge node in Classes 1 (0.964), 2 (1.076), and 4 (0.659), and second in Class 3 (0.738). Knowledge sharing was among the top three bridge nodes in all classes. Organizational commitment uniquely ranked first in Class 3 (0.787) (see Figure 3).

**Table 6:** Top 5 bridge centrality nodes by LCA class.

Rank	Class 1	BS	Class 2	BS	Class 3	BS	Class 4	BS
1	Person-job fit	0.964	Person-job fit	1.076	Org. commitment	0.787	Person-job fit	0.659
2	Knowledge sharing	0.962	Knowledge sharing	0.914	Person-job fit	0.738	Knowledge sharing	0.602
3	Safety performance	0.841	Safety performance	0.788	Knowledge sharing	0.609	Safety performance	0.500
4	Org. commitment	0.756	Challenge demands	0.473	Safety performance	0.604	Org. commitment	0.477
5	Structural resources	0.583	Org. commitment	0.467	Structural resources	0.525	Reducing hindrances	0.297

### Intervention potential: class-specific prioritization

**Figure 4:**
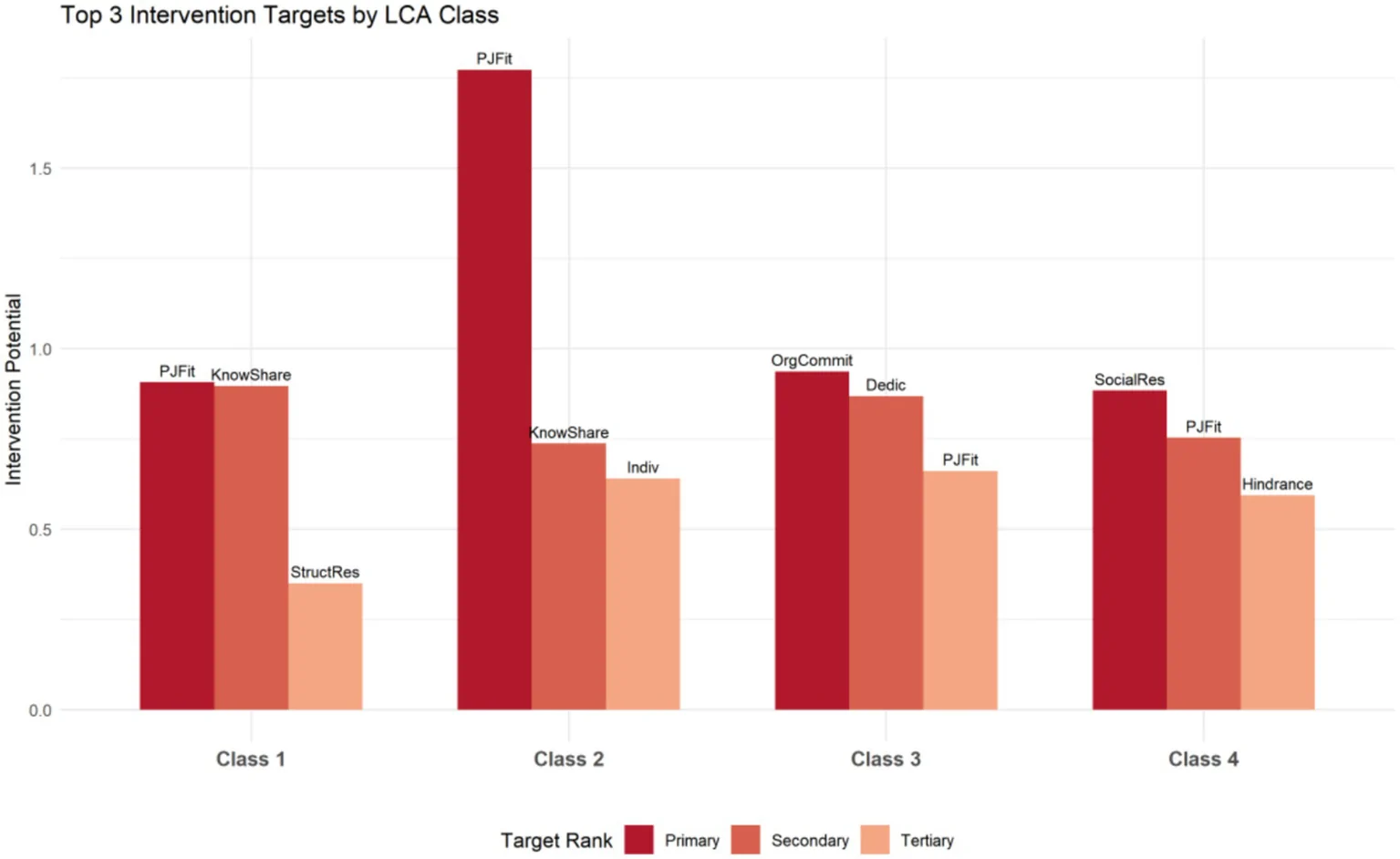
Top 3 intervention targets by LCA class.

A network-based candidate target index (average of standardized EI and bridge strength) was computed (Figure 4; Table 7). In Class 1, person-job fit (1.773), knowledge sharing (1.714), and safety performance (1.352) were top candidate targets. In Class 2, person-job fit (1.365), organizational commitment (0.937), and dedication (0.868). In Class 3, organizational commitment (0.937), person–job fit (0.907), and dedication (0.868). In Class 4, although the network-based candidate target index identified social resources (0.883), person-job fit (0.753), and reducing hindrances (0.594) as top-ranked nodes, the instability of the underlying centrality estimates (CS-EI = 0.050) precludes substantive interpretation of these findings. They are reported for completeness but excluded from the profile-specific intervention recommendations.

**Table 7:** Top 3 candidate targets by LCA class.

Rank	Class 1	Score	Class 2	Score	Class 3	Score	Class 4	Score
1	Person-job fit	1.773	Person-job fit	1.365	Org. commitment	0.937	Social resources	0.883
2	Knowledge sharing	1.714	Org. commitment	0.937	Person-job fit	0.907	Person-job fit	0.753
3	Safety performance	1.352	Dedication	0.868	Dedication	0.868	Reducing hindrances	0.594

### Network accuracy and stability

Nonparametric bootstrap (5,000 samples) indicated adequate edge-weight precision (Supplementary Figures S1–S4). Case-dropping bootstrap CS-coefficients for Expected Influence were 0.594 (Class 1), 0.514 (Class 2), 0.359 (Class 3), and 0.050 (Class 4). Strength CS-coefficients were 0.438, 0.437, 0.517, and 0.050, respectively (Table 8). Notably, the correlation-stability coefficient for both strength and expected influence in Class 4 (Optimal) was 0.050, far below the minimum recommended threshold of 0.25 (Epskamp et al., 2018). This indicates that the centrality ordering for this class is statistically unreliable and should not be used for substantive interpretation. The likely cause is restricted variance due to ceiling effects, because participants in Class 4 scored near the maximum on all 15 dimensions (Ms = 4.85–4.97), resulting in a near-singular covariance matrix that renders edge and centrality estimates unstable. Therefore, centrality, bridge centrality, and intervention potential findings for Class 4 are reported for transparency and completeness but are excluded from substantive discussion and intervention recommendations.

**Table 8:** Correlation-stability (CS) coefficients for centrality indices by LCA class.

Class	CS-Strength	CS-EI
1 (*n* = 534)	0.438	0.594
2 (*n* = 142)	0.437	0.514
3 (*n* = 234)	0.517	0.359
4 (*n* = 337)	0.050	0.050

Values > 0.50 indicate excellent stability; > 0.25 acceptable. CS-EI for Class 4 is below the acceptable threshold, likely due to restricted variance from ceiling effects.

### Robustness of network structures to the tuning parameter

The bridge centrality rankings were invariant to the choice of the EBIC tuning parameter *γ*. Across γ = 0.25, 0.50, and 0.75, person-job fit and knowledge sharing consistently ranked as the top two bridge nodes in Classes 1, 2, and 4, while organizational commitment remained the strongest bridge node in Class 3 (Supplementary Table S1).

### Network comparison across LCA classes

NCTs (500 permutations) revealed significant global strength differences between Class 1 and all other classes (Class 1 vs. 2: *p* = 0.028; Class 1 vs. 3: *p* = 0.002; Class 1 vs. 4: *p* = 0.002), indicating that the job-crafting-driven profile had a uniquely dense network. Significant network structure differences were observed between Class 1 and 4 (*p* = 0.018) and between Class 2 and 3 (*p* = 0.044), suggesting fundamentally different edge-weight patterns between these pairs. No other comparisons reached significance (Table 9).

**Table 9:** Network comparison test (NCT) results across LCA classes.

Comparison	Global strength (1 vs. 2)	*p*	Network structure M	*p*
Class 1 vs. Class 2	7.39 vs. 6.39	**0.028**	0.419	0.106
Class 1 vs. Class 3	7.39 vs. 6.11	**0.002**	0.266	0.174
Class 1 vs. Class 4	7.39 vs. 5.55	**0.002**	0.438	**0.018**
Class 2 vs. Class 3	6.39 vs. 6.11	0.585	0.370	**0.044**
Class 2 vs. Class 4	6.39 vs. 5.55	0.164	0.414	0.331
Class 3 vs. Class 4	6.11 vs. 5.55	0.098	0.278	0.397

Significant values (*p* < 0.05) are bolded. NCT conducted with 500 permutations.

## Discussion

This study applied LCA and network analysis to a sample of 1,247 frontline miners to identify distinct psychological profiles and their associated network structures, bridge nodes, and candidate intervention priorities. Four profiles, namely Job-Crafting-Driven (42.8%), Leadership-Dependent (11.4%), Low-Resource Vulnerable (18.8%), and Optimal (27.0%), exhibited qualitatively different network architectures, suggesting that miner safety is sustained by profile-specific resource configurations rather than a single universal structure.

### Heterogeneity in miners’ psychological networks

The four profiles displayed a clear gradient in network density that paralleled overall psychological resource levels. The Job-Crafting-Driven profile showed the densest network (0.560), whereas the Optimal profile showed the sparsest (0.320). This gradient only partially aligns with the buffering hypothesis (Bakker and Demerouti, 2017). While the Optimal class indeed exhibited the sparsest network, consistent with the notion that high-resource individuals possess redundant protective systems, the Job-Crafting-Driven class, which was not the highest-resource group, displayed the densest interconnectivity. This pattern may reflect a distinct dynamic. Active job crafting, by its nature, involves mobilizing multiple resources simultaneously, which may create denser interconnections among psychological states precisely because these miners are actively engaging with their work environment (Super, 1980). Thus, high interconnectivity among the non-optimal classes may reflect a state of active resource mobilization rather than vulnerability per se. This interpretation is consistent with the density gradient reported by Portoghese et al. (2025), who also observed that latent profiles with intermediate resource levels exhibited denser networks than the highest-resource profile among Italian hospital workers. The Network Comparison Test reinforced this interpretation. The Job-Crafting-Driven profile differed significantly in global strength from the other three profiles, and its network structure differed significantly from that of the Optimal profile. These formal tests confirm that the intermediate profile is not merely a diluted version of the optimal one but represents a qualitatively distinct state characterized by high interconnectivity.

### Person-job fit and knowledge sharing as trans-profile bridge nodes

A central contribution of this study is the identification of person-job fit and knowledge sharing as the most consistent bridge nodes across profiles. Person-job fit ranked first in bridge strength in three of four classes, and knowledge sharing appeared among the top three bridge nodes in all four profiles. This trans-profile stability underscores the strategic importance of these two constructs in maintaining connectivity among the leadership, engagement, crafting, and safety subnetworks. The prominence of person-job fit aligns with foundational work on person-environment fit theory, which posits that perceived congruence between individual capabilities and job demands serves as a meta-resource that facilitates the mobilization of other resources. Knowledge sharing, as a behavioral conduit, has long been recognized as a cornerstone of safety climate, particularly in high-risk industries where tacit knowledge transfer can prevent catastrophic errors. The present study extends this work in two respects: first, by demonstrating that bridge centrality patterns differ by psychological profile rather than being workforce-invariant; and second, by identifying person–job fit and knowledge sharing, constructs central to organizational psychology, as trans-profile bridge nodes, thereby complementing the more clinically oriented bridge variables documented in prior research (Zhao et al., 2026).

An unexpected finding was the consistent appearance of safety performance among the top bridge nodes in all four classes (ranked third in Classes 1, 2, and 4; fourth in Class 3). As the outcome variable, safety performance is conceptually downstream of the other constructs. Its high bridge strength may reflect the possibility that safety behavior, once established, becomes a reinforcing node that connects and stabilizes the broader psychological system, a pattern consistent with reciprocal determinism in social cognitive theory (Bandura, 1986). This interpretation has received indirect support from recent work demonstrating reciprocal relationships between safety participation and psychological wellbeing, and from findings that safety citizenship behavior can enhance subsequent work engagement (Curcuruto and Griffin, 2018). Nonetheless, direct longitudinal evidence of safety performance functioning as a bridge node in a psychological network remains to be established. Alternatively, this finding may be a statistical artifact of the cross-sectional design, in which all variables, including the outcome, are treated symmetrically. Future longitudinal network analyses could clarify whether safety performance genuinely serves a feedback function within the psychological system.

### Profile-specific candidate intervention targets

The network-based candidate target index, which integrates expected influence and bridge strength, revealed that the most promising candidate targets vary by profile. For the Job-Crafting-Driven group, person-job fit and knowledge sharing were the top two targets, suggesting that interventions aimed at optimizing job placement and fostering peer-to-peer knowledge exchange are hypothesized to be particularly efficient, pending empirical verification. For the Leadership-Dependent group, person-job fit again ranked highest, followed by organizational commitment, indicating that these miners may benefit most from improved job matching and enhanced organizational attachment, though this awaits experimental confirmation. For the Low-Resource Vulnerable group, organizational commitment was the primary target, suggesting that building loyalty and a sense of belonging may be the most efficient first step toward systemic improvement, a hypothesis that requires longitudinal testing. For the Optimal group, the highest-ranked nodes in the network-based candidate target index were social resources, person-job fit, and reducing hindrances. However, as noted in the Results, the instability of the centrality estimates for this class (CS-coefficients ≈ 0.05) precludes substantive interpretation of these rankings, and they are not used to inform intervention recommendations.

Translating profile-specific candidate intervention priorities into practice presents implementation challenges that warrant consideration. Identifying which profile a given miner belongs to would require periodic assessment of the 15 dimensions, which could be integrated into existing safety training and health screening routines. Profile classification algorithms based on the current LCA model could streamline this process in future applications. From an organizational perspective, implementing profile-specific interventions would require a shift from uniform safety programs to a stratified approach in which resources are allocated differentially across worker subgroups. While this may entail additional upfront costs, the potential efficiency gains, by targeting the most structurally influential nodes for each profile, could offset these investments. These feasibility considerations remain speculative and should be investigated in future implementation research.

This profile-specific prioritization represents an advance over one-size-fits-all safety interventions. By aligning candidate intervention targets with the underlying network structure of each profile, enterprises can allocate limited resources more efficiently, a principle that resonates with the precision public health approach recently advocated in occupational safety research. Importantly, these cross-sectional findings are hypothesis-generating; the actual effectiveness of the identified candidate targets requires experimental or longitudinal verification.

### Limitations and future directions

Several limitations warrant consideration. First, the cross-sectional design precludes causal inference and the examination of temporal dynamics; future studies should employ intensive longitudinal designs to capture how these networks evolve. Second, the sample was limited to male miners in state-owned intelligent coal mines in Shanxi Province, which may restrict generalizability to other regions, mine types, and gender-diverse workforces. The preponderance of male participants (88.9%) also precludes the examination of gender differences. Third, the correlation-stability coefficient for expected influence in the Optimal profile was only 0.050, far below the acceptable threshold of 0.25. This low stability likely reflects restricted variance due to ceiling effects, when almost all miners in a profile score near the maximum on every dimension, the estimated edges become unstable. Thus, the centrality findings for the Optimal profile should be interpreted with caution, and future research should consider oversampling high-functioning miners or employing more robust regularization techniques. Fourth, the present study relied on self-report measures, which are subject to common-method variance and social desirability. Although CFA-based tests suggested that common method bias was unlikely to threaten the validity of the findings, future work should incorporate objective safety records and multisource assessments.

## Conclusion

By identifying four distinct psychological profiles and their corresponding network architectures, this study demonstrates that miner safety is sustained by profile-specific resource configurations rather than a single universal structure. A clear density gradient emerged across profiles, with the Job-Crafting-Driven class exhibiting the highest interconnectivity and the Optimal class the lowest. Network Comparison Tests confirmed that these profiles represent structurally distinct psychological systems, not merely variations on a theme. Person–job fit and knowledge sharing consistently emerged as trans-profile bridge nodes, and profile-specific candidate intervention priorities were identified through an integrated network-based candidate target index. Theoretically, the study demonstrates that a person-centered network approach can reveal heterogeneity in psychological architecture that would be obscured in traditional variable-centered analyses, showing that the same construct can serve qualitatively different structural roles across latent profiles. Practically, the findings suggest that safety management in mining should move beyond one-size-fits-all interventions toward stratified programs that prioritize different nodes for different worker profiles, such as person–job fit and knowledge sharing for the majority Job-Crafting-Driven profile, and organizational commitment for the Low-Resource Vulnerable profile. These recommendations await experimental and longitudinal validation but offer a data-driven starting point for designing profile-sensitive safety programs.

## Data Availability

The datasets presented in this article are not readily available because the data presented in this study are available on request from the corresponding author. The data are not publicly available due to privacy restrictions and the sensitive nature of information pertaining to coal mining enterprises. Requests to access the datasets should be directed to pangxiaohua@czmc.edu.cn.
