# Correction: Identifying profile-specific candidate targets for miner safety: a latent class and network analysis of psychological resources

**DOI:** 10.3389/fpsyg.2026.2030883

**Published:** 2026-10-28

**Authors:** Xiaohua Pang, Jizu Li, Xuehua Xu

**Affiliations:** 1 School of Economics and Management, Taiyuan University of Technology, Taiyuan, China; 2 Department of Mental Health, Changzhi Medical College, Changzhi, China

**Keywords:** miner safety, psychological resources, latent class analysis, psychological network analysis, profile-specific intervention targets, person-job fit, knowledge sharing

In the published article, an incorrect grant number was provided for [23YJA630054]. The correct grant number is [22YJC630194]. The correct **Funding** statement is as below:

The author(s) declared that financial support was received for this work and/or its publication. This work was supported by China Ministry of Education Humanities and Social Sciences Planning Fund Project (Grant No. 22YJC630194).

The original version of this article has been updated.

