# Supplementary material for: Identifying profile-specific candidate targets for miner safety: a latent class and network analysis of psychological resources
Source: Front Psychol. 2026 Jul 9;17:1877732. doi: 10.3389/fpsyg.2026.1877732 (PMC13391247; doi:10.3389/fpsyg.2026.1877732)
Supplement: Supplementary file 1 [file Supplementary_file_1.docx]

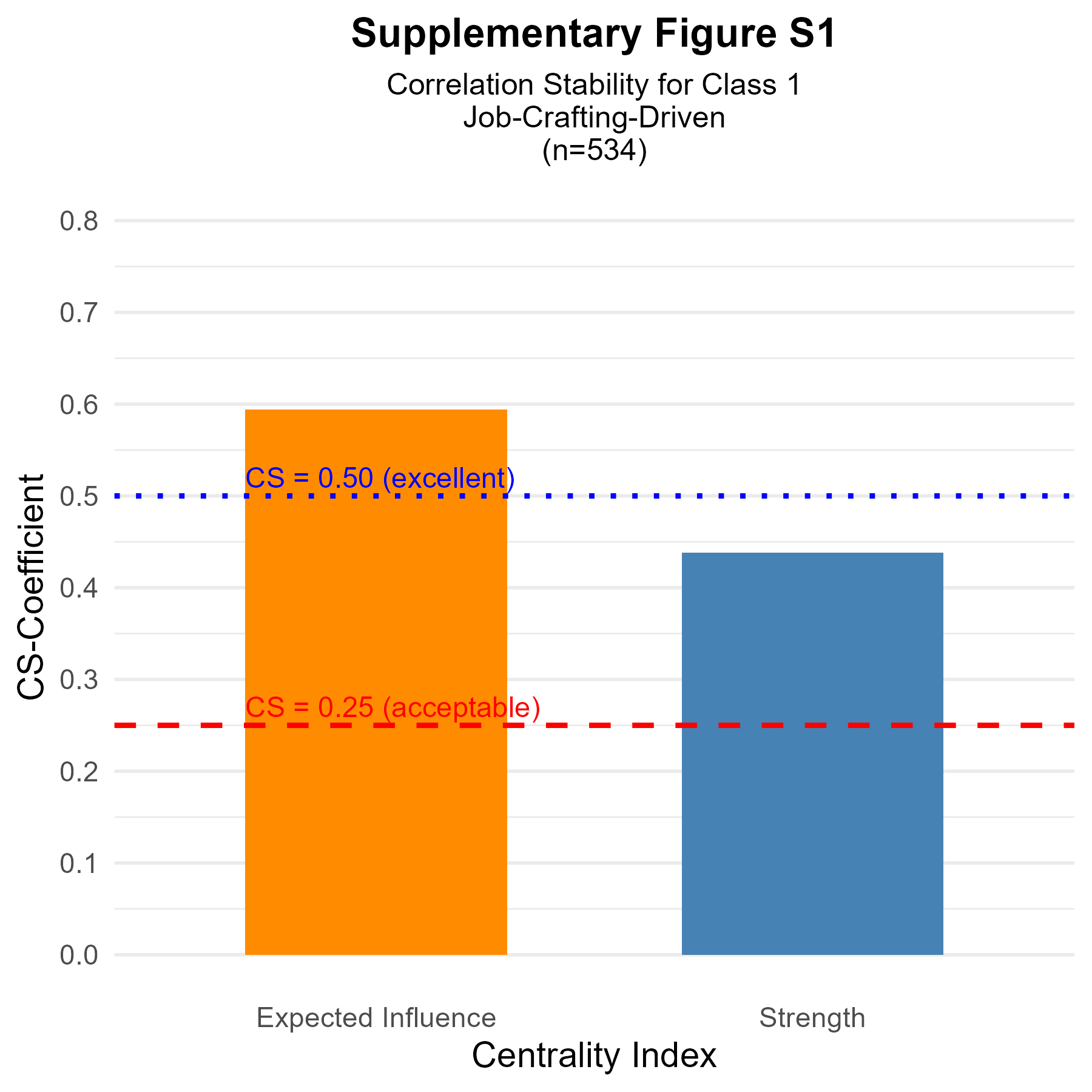


**Supplementary Figure S1:** CS-coefficients for Class (Job-Crafting-Driven)
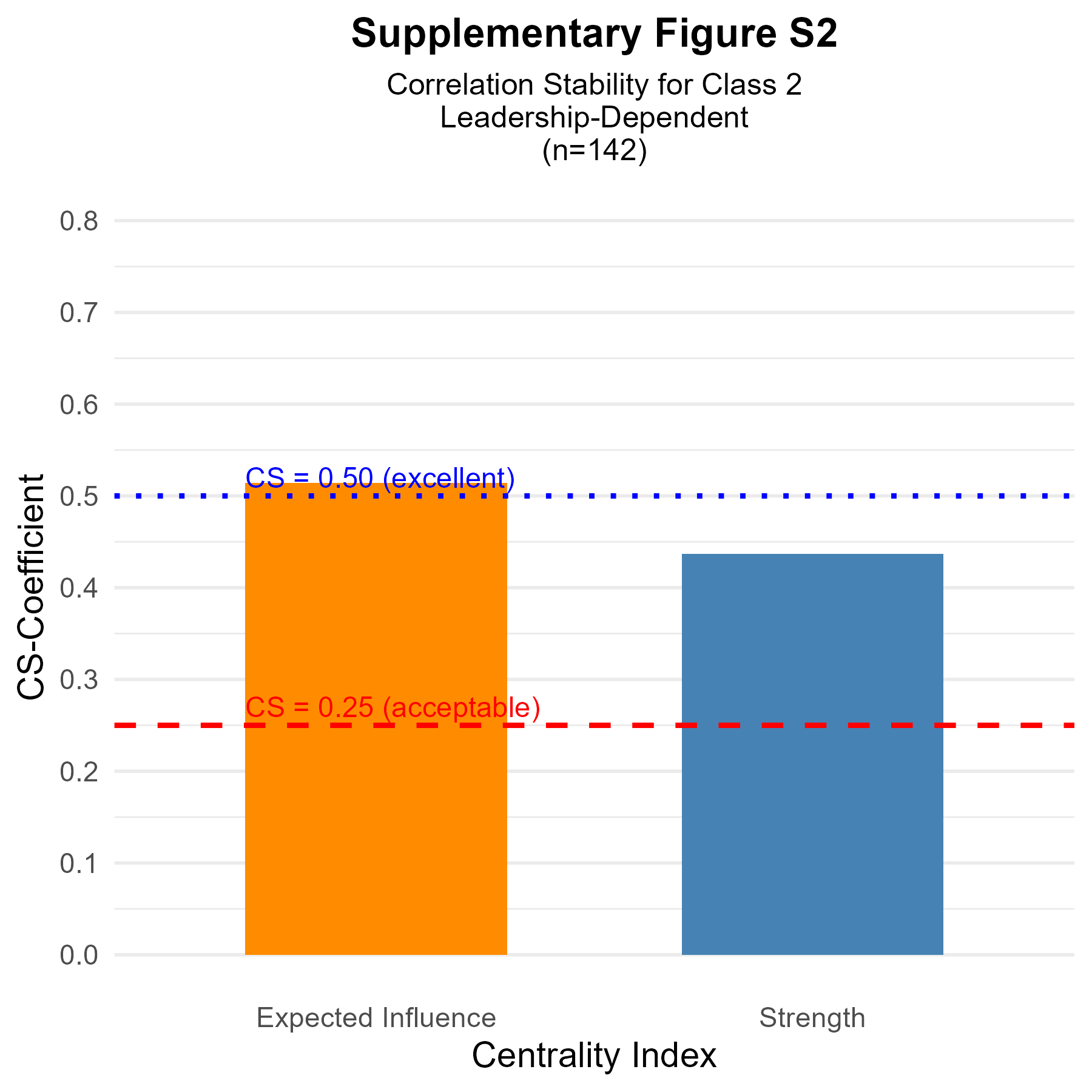


**Supplementary Figure S2:** CS-coefficients for Class 2 (Leadership-Dependent)


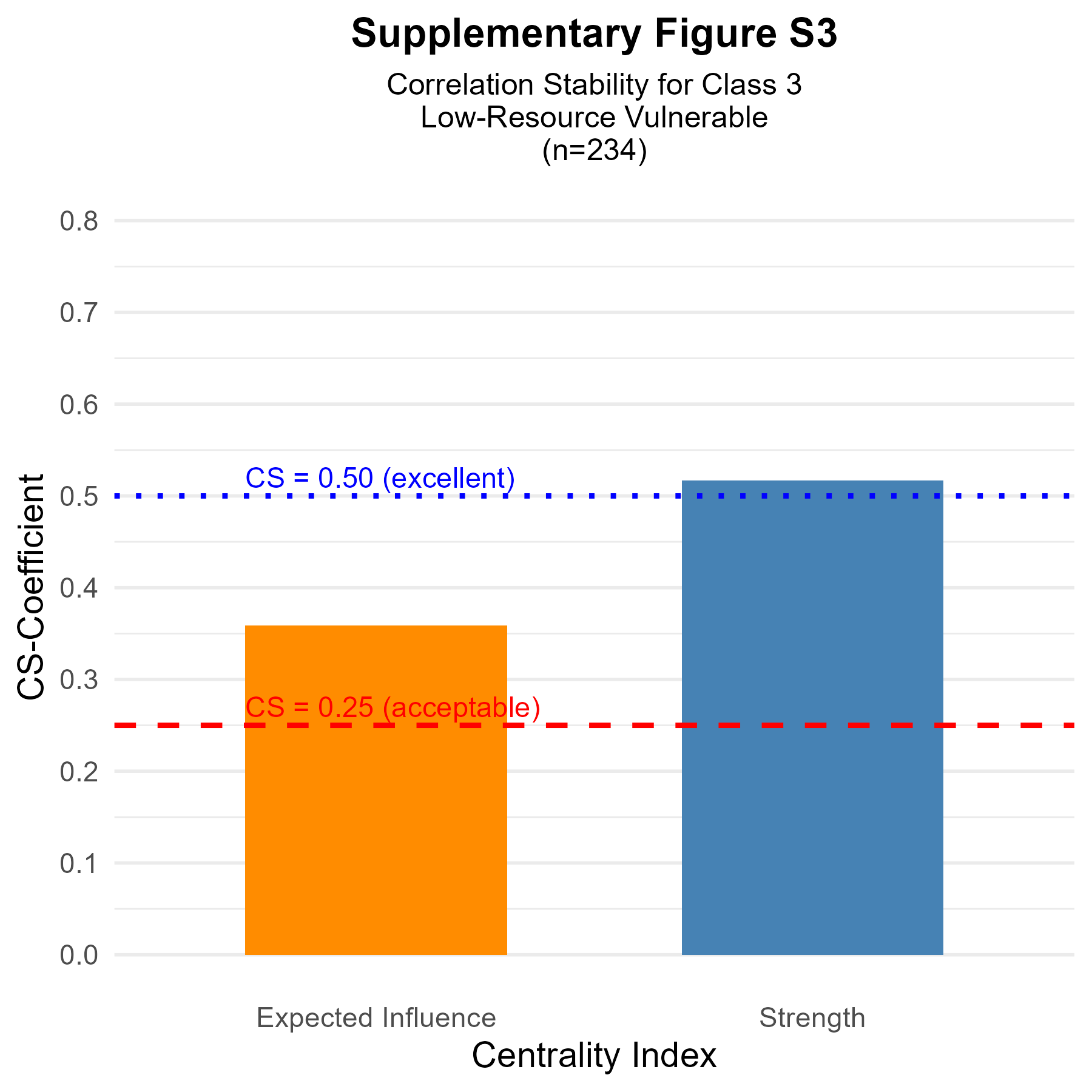


**Supplementary Figure S3:** CS-coefficients for Class 3 (Low-Resource Vulnerable)


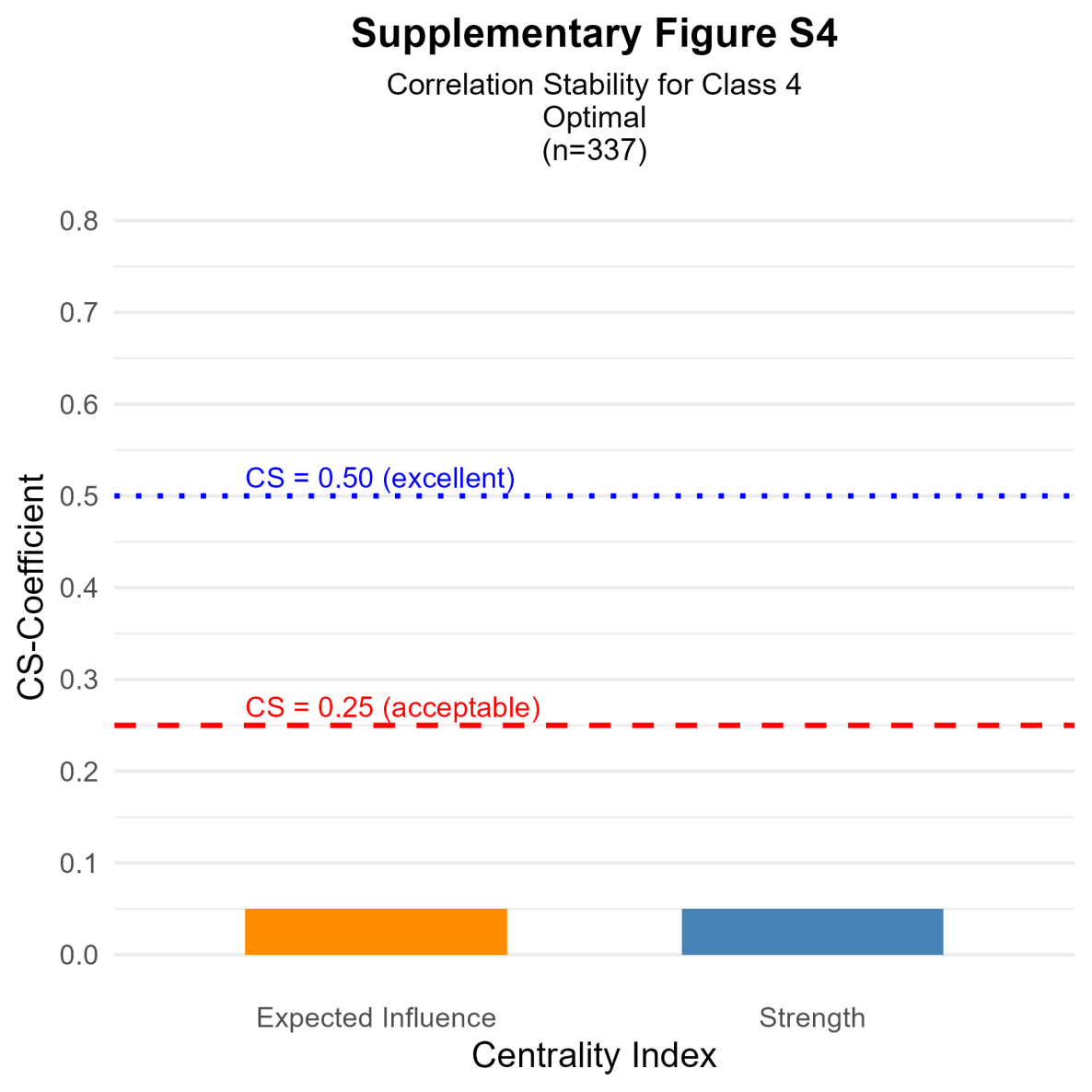


**Supplementary Figure S4:** CS-coefficients for Class 4 (Optimal)
