# Supplementary material for: Identifying profile-specific candidate targets for miner safety: a latent class and network analysis of psychological resources
Source: Front Psychol. 2026 Jul 9;17:1877732. doi: 10.3389/fpsyg.2026.1877732 (PMC13391247; doi:10.3389/fpsyg.2026.1877732)
Supplement: Supplementary file 2 [file Table_1.docx]

**Supplementary Materials**

**Supplementary Table S1.** LCA Model Fit Indices for 2- to 6-Class Solutions

| **Classes** | **AIC** | **BIC** | **ΔBIC** |
| --- | --- | --- | --- |
| 2 | 18,556.34 | 18,797.38 | — |
| 3 | 16,174.61 | 16,538.73 | 2,258.65 |
| **4** | **15,459.95** | **15,947.16** | **591.57** |
| 5 | 15,053.07 | 15,663.36 | 283.80 |
| 6 | 14,806.49 | 15,539.86 | 123.50 |

***Note.*** BIC = Bayesian Information Criterion; ΔBIC = improvement in BIC from the *k*-class to the (*k*+1)-class solution. The four-class solution (bolded) was selected based on the Elbow rule and profile interpretability.

**Supplementary Table S2** Global Properties of Gaussian Graphical Models Estimated Within Each LCA Class

| Property | Class 1 (Job‑Crafting‑Driven) | Class 2 (Leadership‑Dependent) | Class 3 (Low‑Resource Vulnerable) | Class 4 (Optimal) |
| --- | --- | --- | --- | --- |
| n | 534 | 142 | 234 | 337 |
| Density | 0.560 | 0.364 | 0.391 | 0.320 |
| Non‑zero edges | 63 | 41 | 44 | 36 |
| Modularity (Q) | 0.398 | 0.549 | 0.507 | 0.512 |

*Note.*Networks were estimated using the graphical LASSO with extended Bayesian information criterion (EBICglasso; γ = 0.5). Density is the proportion of non‑zero edges out of 105 possible undirected edges. Modularity (Q) was obtained via the Louvain community detection algorithm applied to the absolute weight matrix; higher values indicate clearer separation between predefined communities (transformational leadership, work engagement, safety performance, person–job fit, job crafting, knowledge sharing, organizational commitment).
