# Supplementary material for: Identifying profile-specific candidate targets for miner safety: a latent class and network analysis of psychological resources
Source: Front Psychol. 2026 Jul 9;17:1877732. doi: 10.3389/fpsyg.2026.1877732 (PMC13391247; doi:10.3389/fpsyg.2026.1877732)
Supplement: Supplementary file 3 [file Table_2.docx]

**Supplementary Materials**

**Supplementary Table S1.**Bridge Centrality Rankings of Person–Job Fit and Knowledge Sharing Across γ Values

| Class | γ = 0.25 | γ = 0.50 | γ = 0.75 |
| --- | --- | --- | --- |
|  | PJF / KS | PJF / KS | PJF / KS |
| 1 | 1st / 2nd | 1st / 2nd | 1st / 2nd |
| 2 | 1st / 2nd | 1st / 2nd | 1st / 2nd |
| 3 | 2nd / 3rd | 2nd / 3rd | 2nd / 3rd |
| 4 | 1st / 2nd | 1st / 2nd | 1st / 2nd |

*Note.* PJF = Person–Job Fit; KS = Knowledge Sharing. Rankings are based on bridge strength.
